# Inhibition of tanshinone IIA on renin activity protected against osteoporosis in diabetic mice

**DOI:** 10.1080/13880209.2020.1738502

**Published:** 2020-03-22

**Authors:** Jingjing Zhang, Zixuan Cai, Min Yang, Lijuan Tong, Yan Zhang

**Affiliations:** School of Pharmacy, Nantong University, Nantong, Jiangsu, PR China

**Keywords:** Angiotensin II, diabetic osteoporosis, renin, *Salvia miltiorrhiza*, HEK-293 cells

## Abstract

**Context:**

*Salvia miltiorrhiza* Bge. (Labiatae) (SMB) is applied clinically for management of diabetic osteoporosis in China, and research results has suggested its potential action on renin–angiotensin system (RAS).

**Objective:**

This study screens and explores naturally occurring bioactive constituents from the root of SMB acting on renin activity and evaluates its osteoprotective efficacy in diabetic mice.

**Materials and methods:**

Human embryonic kidney (HEK) 293 cells, engineered to express human renin, were used as an *in vitro* model to identify bioactive compound, tanshinone IIA, inhibiting renin activity. The C57BL/6 mice (*n* = 10 in each group) with diabetes induced by streptozotocin (STZ) were intraperitoneally injected with tanshinone IIA (10 and 30 mg/kg). The mice without STZ treatment and the diabetic mice treated with aliskiren were used as non-diabetic control and positive control, respectively.

**Results:**

Tanshinone IIA was found to display inhibitory effects on renin activity of HEK-293 cells; moreover, it down-regulated protein expression of ANG II in human renin-expressed HEK-293 cells. Treatment of diabetic mice with tanshinone IIA with both doses could significantly decrease ANG II level in serum (from 16.56 ± 1.70 to 10.86 ± 0.68 and 9.14 ± 1.31 pg/mL) and reduce ANG II expression in bone, consequently improving trabecular bone mineral density and micro-structure of proximal tibial end and increasing trabecular bone area of distal femoral end in diabetic mice.

**Conclusions:**

This study revealed beneficial effects of tanshinone IIA on bone of diabetic mice, and potentially suggested the application of *Salvia miltiorrhiza* in the treatment of osteoporosis and drug development of tanshinone IIA as a renin inhibitor.

## Introduction

The incidence of type 1 diabetes (T1D) with chronic hyperglycaemic state has been globally rising during the past decades (Starup-Linde and Vestergaard 2015). Besides the well-evident diabetic complications such as neuropathy, nephropathy and retinopathy, T1D is also characterized by poor bone quality such as osteoporosis, even though the affection of type 2 diabetes mellitus on bone mineral density (BMD) is controversial (Adil et al. 2015; Siddapur et al. 2015). The increase in the prevalence of osteoporosis induced by T1D has prompted researchers to explore novel biological reagents for the management of diabetic osteoporosis. Currently, there is no consensus about therapeutic intervention for bone disorders induced by hyperglycaemia (Zhukouskaya et al. 2015). Thus, the treatment for bone injuries associated with diabetes lies in a core role in treating diabetic complications.

Angiotensin II (ANG II) is produced from ANG I by angiotensin-converting enzyme (ACE), and ANG I is derived from angiotensinogen by renin. The emerging evidence revealed the expression of renin–angiotensin system (RAS) components in skeletal system (Zhang Y et al. 2016a, 2016b). ANG II, active peptide within RAS, plays a pathological role in inducing the development of osteopenia and osteoporosis through activating osteoclastogenesis and inhibiting osteogenesis by binding to type 1 receptor (AT1R) (Shimizu et al. 2008; Nakai et al. 2013). *In vivo* and *in vitro* studies showed that the increased activity of skeletal RAS, especially the over-activation of the renin/ANG II/AT1R signalling at high glucose state, was detrimental to bone tissue (Zhang Y et al. 2014, 2016a, 2016b; Li et al. 2015; Yamamoto et al. 2015). Since renin is the rate-limiting enzyme of the RAS cascade (Zhang Y et al. 2012), the renin inhibitor like aliskiren exerted beneficial effects on skeletal system in animal studies (Zhang FY et al. 2014; Zhang Y et al. 2016a, 2016b). While the therapeutic reagents derived from natural products and traditional herbs acting on the renin/ANG II/AT1R pathway are very limited.

*Salvia miltiorrhiza* Bge. (Labiatae, SMB), distributed in the Southeast Asia, could promote blood circulation and remove blood stasis according to theory of traditional Chinese medicine (Zhang et al. 2006). In clinical practice, it is always as one main component applied in herbal formula for management of diabetic osteoporosis (Ma et al. 2016). Most importantly, Qishenyiqi dropping pill including SMB as monarch herb could attenuate myocardial fibrosis through suppressing the RAS pathway (Wang et al. 2015), suggesting a potential action of SMB on RAS cascade. So far the reported functional compounds found in SMB mainly include tanshinone IIA, succinic acid, ferulic acid, caffeic acid and danshinolic acid (Ni et al. 2019).

Thus, the present study aimed to first screen and explore the potential candidate modulating RAS cascade such as renin activity and ANG II production, and then investigate the ameliorative effects of the candidate compound on bone damages associated with hyperglycaemia induced by streptozotocin (STZ) injection in mice.

## Materials and methods

### Cell culture and treatment

Human embryonic kidney (HEK) 293 cells were engineered to express human renin (Genomeditech, Shanghai, China). The stably transfected cells were cultured in Dulbecco’s modified Eagle medium (Biosera, Nuaille, France) containing 10% foetal bovine serum at 37 °C in a humidified atmosphere of 95% air and 5% CO_2_, and treated with vehicle (DMSO) or aliskiren (10^−6^ M, Sigma, St. Louis, MO) or well-known active compounds (National Institutes for Food and Drug Control, Beijing, China) in *Salvia miltiorrhiza*, including tanshinone IIA, succinic acid, ferulic acid, caffeic acid and danshinolic acid, with different final concentrations (10^−8^, 10^−7^, 10^−6^ M). After drug treatment for 24 h, cell protein was isolated by RIPA lysis buffer (Beyotime, Beijing, China) for further analysis on renin activity and ANG II protein expression.

### Animals and treatments

C57BL/6 mice (Slac Laboratory, Shanghai, China) were housed in environmentally controlled central animal facilities and exposed to a 12 h light/dark cycle. Eight-week-old male mice were made diabetic by intraperitoneal (ip) injection of freshly prepared STZ (40 mg/kg) dissolved in citrate buffer (10 mM, pH 4.2) for five consecutive days. Two weeks after STZ injection, the diabetic mice were randomly separated into four groups and treated, respectively, with vehicle (D; ip injection, three times each week), aliskiren (2 mg/kg, D + Ali), tanshinone IIA at low dose (10 mg/kg, D + LT) and high dose (30 mg/kg, D + HT) with ip injection (three times each week). Non-diabetic (ND) mice were as control group without STZ or any drug treatment and corn oil was used as drug vehicle. Fasting blood glucose level was monitored with the CONTOUR blood glucose-monitoring system (Bayer, Leverkusen, Germany). All mice were killed after 8 weeks of drug treatment. Serum, tibias and femurs were immediately harvested for a variety of biochemical and histological analyses. Animal experiments were conducted in accordance with the NIH guide for the care and use of laboratory animals. The animal study protocol was approved by the Institutional Animal Care and Use Committee at Nantong University.

### Micro-computed tomography (Micro-CT) scanning and analysis

The tibias were scanned with a high-resolution micro vivaCT 40 system (Scanco Medical, Wangen-Brüttisellen, Switzerland). The detection parameters and the separation of trabecular bone at tibial proximal metaphysis were set and performed, respectively, as previously described (Zhang Y et al. 2016a, 2016b). 3-Dimensional (3D) images were obtained and the quantitative parameters as the followings were recorded: (1) BMD over total volume (BMD/TV); (2) connectivity density (Conn.D); (3) bone volume over total volume (BV/TV); (4) structure model index (SMI).

### Measurement for renin activity and angiotensin II level

Renin Assay Kit (Sigma, St. Louis, MO) was used to determine the activity of renin in cell protein. ANG II level in mice serum was assessed by commercially available enzyme-linked immunosorbent assay (ELISA) kit (Sigma, St. Louis, MO) according to the manufacturer’s instruction.

### Histological staining on trabecular bone

The femurs were fixed, decalcified in EDTA glycerol solution, and embedded in paraffin. Serial sections of 3 µm were cut on a rotary microtome. Haematoxylin and eosin staining was performed on the distal metaphysis of femur. Trabecular bone quantity expressed as trabecular bone area over total area (BA/TA) was measured using OsteoMeasure system (OsteoMetrics Inc., Decatur, GA).

### Immunostaining on trabecular bone

The femoral paraffin sections were first boiled in 10 mM Na citrate solution (pH 6.0) for 10 min and stained with mouse monoclonal antibody (Santa Cruz, Paso Robles, CA) against ANG II, followed by incubation with goat anti-mouse IgG (Jackson ImmunoResearch, West Grove, PA). Nuclei were counterstained with 4,6-diamidino-2-phenylindole (DAPI). Images were captured under a microscope (Leica DM 2500, Wetzlar, Germany). The proportional area for ANG II labelling was determined for each image by densitometric scanning with NIH ImageJ software (NIH Image J system, Bethesda, MD).

### Immunoblotting

The protein concentration in cell lysates was determined using a Bio-Rad Protein Assay kit (Bio-Rad, Hercules, CA). Samples containing 20 μg of protein were separated on 10% SDS-PAGE gel, transferred to nitrocellulose membranes (Bio-Rad Laboratories, Hercules, CA). After saturation with 5% (w/v) non-fat dry milk in TBS and 0.1% (w/v) Tween 20 (TBST), the membranes were incubated with mouse anti-ANG II monoclonal antibody (Santa Cruz, Paso Robles, CA). After three washes with TBST, membranes were incubated with secondary immunoglobulins conjugated to IRDye 800CW Infrared Dye (LI-COR, Lincoln, NE). Blots were visualized by the Odyssey Infrared Imaging System (LI-COR Biotechnology, Lincoln, NE). Signals were densitometrically assessed (Odyssey Application Software version 3.0; LI-COR Biotechnology, Lincoln, NE) and normalized to β-actin signal using the mouse monoclonal anti-β-actin antibody (Sigma, St. Louis, MO).

### Statistical analysis

The data from these experiments were reported as mean ± standard error of mean (SEM) for each group. The statistical analysis was performed using PRISM version 4.0 (GraphPad, La Jolla, CA). Inter-group differences were analysed by one-way ANOVA, and followed by Tukey’s multiple comparison test as a *post hoc* test to compare the group means if overall *p* < 0.05. The difference with *p* value of less than 0.05 was considered statistically significant.

## Results

### Screening of compounds from SMB on renin activity inhibition

**Figure 1.**
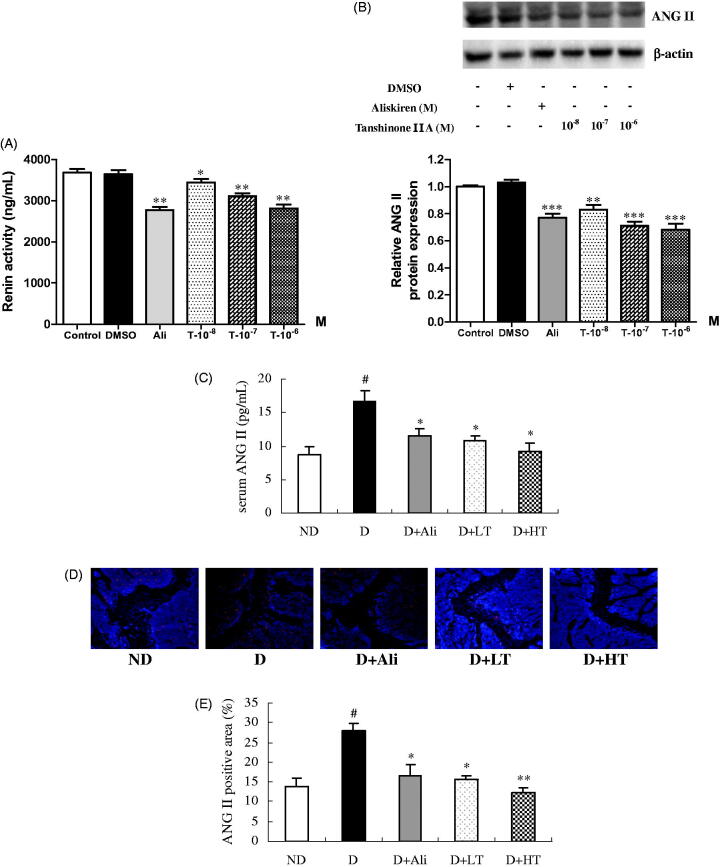
Renin activity (A) and angiotensin II (ANG II) protein expression (B) in human renin-transfected HEK-293 cells as well as ANG II level in serum (C) and ANG II expression in distal metaphysis of femur (D). The cells were treated with vehicle (DMSO), renin inhibitor aliskiren (Ali, 10^−6^ M) or tanshinone IIA (10^−8^ M, 10^−7^ M, 10^−6^ M) for 24 h. Values were expressed as means ± SEM with at least three independent experiments. *p < 0.05, **p < 0.01, ***p < 0.001, vs. DMSO group. The diabetic mice treated with vehicle (D), aliskiren (D + Ali, 2 mg/kg), and low (D + LT, 10 mg/kg) or high (D + HT, 30 mg/kg) dose of tanshinone IIA for 8 weeks. Immunostaining for ANG II was performed on trabecular bone at distal femoral end and the positive signal was shown by red colour. (E) Average proportional area of positive staining for ANG II. Values were expressed as means ± SEM, *n* = 10. ^#^*p* < 0.05, vs. non-diabetic (ND) group. *p < 0.05, **p < 0.01, vs. D group.

Incubation of human renin-expressed HEK-293 cells with tanshinone IIA at three doses significantly inhibited renin activity as compared to that in the DMSO group (Figure 1(A)), but other compounds which are also main functional components in SMB, such as succinic acid, ferulic acid, caffeic acid and danshinolic acid, did not influence renin activity of HEK-293 cells highly expressing human renin (data not shown).

### Effects of tanshinone IIA on ANG II expression

As similar to the renin inhibitor aliskiren (Figure 1(B), *p* < 0.001), treatment of HEK-293 cells with tanshinone IIA at 10^−8^ M (*p* < 0.01), 10^−7^ M (*p* < 0.001) and 10^−6^ M (*p* < 0.001) significantly down-regulated protein expression of ANG II, suggesting tanshinone IIA could effectively diminish ANG II expression by inhibiting renin activity.

### Effects of tanshinone IIA on ANG II level in serum and bone of diabetic mice

The present study clearly showed that the serum level of ANG II (Figure 1(C)) and the positive staining on ANG II at distal metaphysis of femur (Figure 1(D)) were both dramatically decreased in diabetic mice in response to treatment with aliskiren or tanshinone IIA in comparison with those of vehicle-treated diabetic mice. The quantitative data (Figure 1(E)) showed that the treatment with either aliskiren (*p* < 0.05) or tanshinone IIA (*p* < 0.05) mitigated the proportional positive area of ANG II in trabecular bone of diabetic mice.

### Effects of tanshinone IIA on trabecular bone of diabetic mice

**Figure 2.**
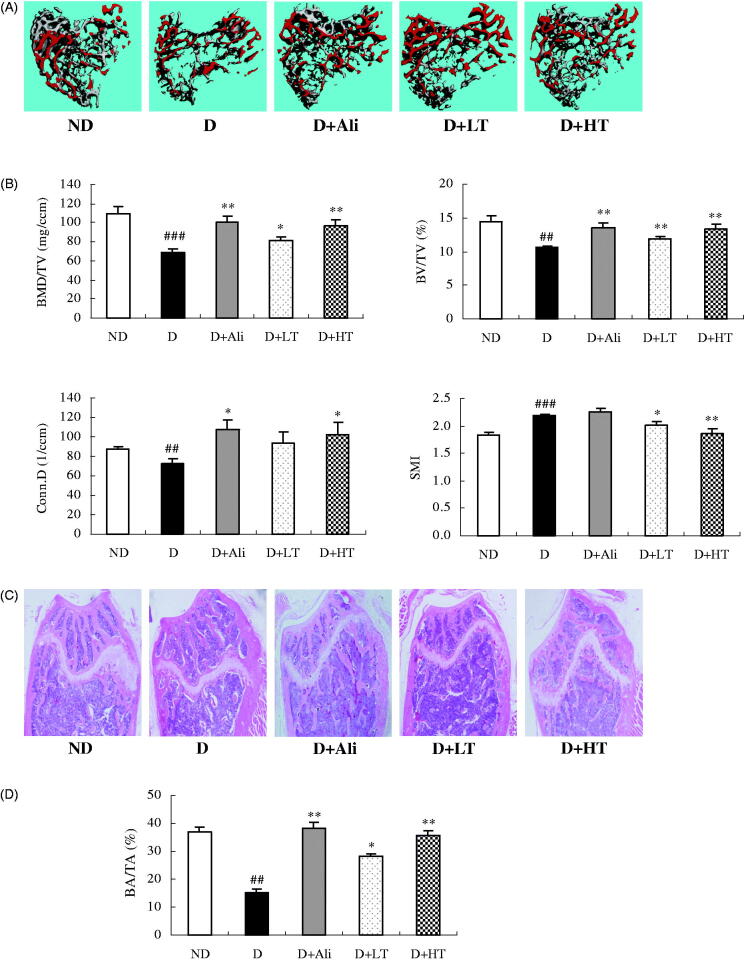
Micro-computed tomography analysis and haematoxylin and eosin (HE) staining on trabecular bone in non-diabetic mice (ND) and diabetic mice treated with vehicle (D), aliskiren (D + Ali, 2 mg/kg), and low (D + LT, 10 mg/kg) or high (D + HT, 30 mg/kg) dose of tanshinone IIA for 8 weeks. (A) Representative three-dimensional image of the trabecular bone at proximal tibial metaphysis. (B) Trabecular bone biological parameters. (C) Representative HE staining images of the trabecular bone at distal femoral metaphysis. (D) Quantitative trabecular bone area over total area (BA/TA). BMD/TV: bone mineral density over total volume; Conn.D: connectivity density; BV/TV: bone volume over total volume; SMI: structure model index. Values were expressed as means ± SEM, *n* = 10. ^##^*p* < 0.01, ^###^*p* < 0.001, vs. ND group. *p < 0.05, **p < 0.01, vs. D group.

As expected, the profiles of 3D images (Figure 2(A)) clearly demonstrated the loss of trabecular bone mass and the breakage of cancellous bone at proximal metaphysis of tibia of diabetic mice, and the 3D bone biological parameters (Figure 2(B)) quantitatively reflected the significant reduction in BMD (*p* < 0.001), trabecular BV/TV (*p* < 0.01) and connectivity density (*p* < 0.01) as well as the marked elevation in SMI (*p* < 0.001) as compared to those of ND control. The treatment of diabetic mice with tanshinone IIA for 8 weeks dramatically improved bone mass of trabecular bone and reversed the changes of biological parameters at proximal tibial head as well as enhanced trabecular BA/TA at distal femoral end assessed by HE staining (Figure 2(C,D)), indicating the potential therapeutic efficacy of tanshinone IIA, even SMB, on diabetic osteoporosis.

## Discussion

The RAS plays a crucial role in controlling plasma volume and blood pressure in body. While the RAS is far more complex than originally thought, much is now known about this system and the wide biological effects of angiotensin (Williams 2016); therefore, it stimulates the development of therapies targeting the various proteins within RAS and hence being implicated in treatment of diseases. The first among these treatments was the angiotensin-converting enzyme inhibitors (ACEIs), followed by the ANG II receptor blockers (ARBs), while the major problem associated with the application of these drugs is the compensatory rise of renin due to the disruption of the feedback inhibition of renin production (Zhang et al. 2008). Additionally, given the contradictory results about the effects of RAS inhibitors including ACEIs and ARBs on skeletal system in previous animal and clinical studies (Kwok et al. 2012; Zhang YF et al. 2012; Zhang Y et al. 2014; Yang et al. 2016), and renin inhibitor aliskiren consistently exerted protection from bone loss of osteoporotic animals (Zhang FY et al. 2014; Zhang Y et al. 2016a, 2016b), it might be a challenge in exploring bioactive ingredient targeting renin activity. Since renin is the rate-limiting enzyme of the RAS (Zhang Y et al. 2016a, 2016b), tremendous research efforts have been performed to identify active renin inhibitors and some drug candidates have entered into clinical trials (Sun et al. 2015). However, to date, only aliskiren was approved. In this study, we are keen to identify functional compound from SMB acting on renin activity and investigate its osteoprotective effects in diabetic mice.

The engineered human renin-expressing HEK-293 cell was used as an *in vitro* model for screening inhibitory effects of compounds from SMB on renin activity and clarifying the regulation of tested compound on RAS cascade. The present study clearly demonstrated the suppression of tanshinone IIA on renin activity of HEK-293 cell highly expressing human renin. The exact mechanism involved in molecular binding and biological cascade response between tanshinone IIA and renin protein need to be further identified. Previous studies have demonstrated that some natural products like salidroside (Chen et al. 2018) and tetrahydroxy stilbene glucoside (Zhang et al. 2019) could affect tissue ANG II level, while this study is the first to explore and report that the naturally occurring product tanshinone IIA from SMB could target renin activity, which, at least partially, explained that SMB could reduce ANG II-stimulated collagen synthesis in cardiac fibroblasts (Ling et al. 2009).

Tanshinone IIA, one of abundant constituents in SMB, has been shown to display antioxidant and anti-inflammatory effects in types of experimental disease models (Gong et al. 2019) and widely demonstrated to produce cardioprotective effects (Zhang Z et al. 2016). To further elucidate the modulation of tanshinone IIA on RAS cascade in bone tissue and the potential improvement on bone damages associated with hyperglycaemia, the STZ-injected diabetic mice were ip administered with tanshinone IIA for 8 weeks. The results suggested that tanshinone IIA was able to decrease *in vivo* circulating and skeletal ANG II level by potentially targeting renin. However, tanshinone IIA did not alter the fasting blood glucose level of diabetic mice (data not shown), indicating the therapeutic effects resulting from the drug treatment was unlikely to be mediated through targeting pancreas or by repressing hyperglycaemia.

Since high renin activity and enhanced ANG II expression contributed to the development of bone deteriorations associated with hyperglycaemia (Chen et al. 2018; Zhang et al. 2019), the beneficial effects of tanshinone IIA on bone tissue was evaluated in diabetic mice. Treatment of diabetic mice with tanshinone IIA markedly increased trabecular BMD and improved trabecular bone micro-architecture as well as enhanced trabecular bone area, in accordance with that tanshinone IIA exerted bone-sparing function in ovariectomized mice (Cheng et al. 2018) and rats (Wang et al. 2019).

## Conclusions

The present study determined that tanshinone IIA could decrease the production of ANG II by inhibiting renin activity, consequently protecting mice with hyperglycaemia from bone deteriorations. The exact interaction between chemical molecule of tanshinone IIA and renin protein requires to be further illustrated, and the potential of this natural product in treating other tissue injuries due to over-activity of tissue RAS remains elusive.
